# A Powerful Method for Transcriptional Profiling of Specific Cell Types in Eukaryotes: Laser-Assisted Microdissection and RNA Sequencing

**DOI:** 10.1371/journal.pone.0029685

**Published:** 2012-01-26

**Authors:** Marc W. Schmid, Anja Schmidt, Ulrich C. Klostermeier, Matthias Barann, Philip Rosenstiel, Ueli Grossniklaus

**Affiliations:** 1 Institute of Plant Biology and Zürich-Basel Plant Science Center, University of Zürich, Zürich, Switzerland; 2 Institute of Clinical Molecular Biology, Christian-Albrechts University, Kiel, Germany; Michigan State University, United States of America

## Abstract

The acquisition of distinct cell fates is central to the development of multicellular organisms and is largely mediated by gene expression patterns specific to individual cells and tissues. A spatially and temporally resolved analysis of gene expression facilitates the elucidation of transcriptional networks linked to cellular identity and function. We present an approach that allows cell type-specific transcriptional profiling of distinct target cells, which are rare and difficult to access, with unprecedented sensitivity and resolution. We combined laser-assisted microdissection (LAM), linear amplification starting from <1 ng of total RNA, and RNA-sequencing (RNA-Seq). As a model we used the central cell of the *Arabidopsis thaliana* female gametophyte, one of the female gametes harbored in the reproductive organs of the flower. We estimated the number of expressed genes to be more than twice the number reported previously in a study using LAM and ATH1 microarrays, and identified several classes of genes that were systematically underrepresented in the transcriptome measured with the ATH1 microarray. Among them are many genes that are likely to be important for developmental processes and specific cellular functions. In addition, we identified several intergenic regions, which are likely to be transcribed, and describe a considerable fraction of reads mapping to introns and regions flanking annotated loci, which may represent alternative transcript isoforms. Finally, we performed a *de novo* assembly of the transcriptome and show that the method is suitable for studying individual cell types of organisms lacking reference sequence information, demonstrating that this approach can be applied to most eukaryotic organisms.

## Introduction

The development of multicellular organisms is controlled by distinct cell fate decisions, which are largely mediated through the establishment of cell- or tissue-specific gene expression patterns. Spatially and temporally resolved information on gene expression patterns facilitate the identification of regulatory networks of gene activity that underly cell differentiation and functional specification. However, transcriptional profiling of specific cell types requires their isolation from an often heterogenic tissue and the determination of the transcriptome, preferentially with high resolution and completeness from ultra-low amounts of RNA (down to single cell resolution).

One method used successfully for the transcriptional profiling of distinct cell types is laser-assisted microdissection (LAM) in combination with DNA microarrays (examples [1], [2] in human and [3]–[6] in plants). LAM allows the isolation of individual cells directly from the surrounding tissue based on histological identification with little cross-contamination (independently validated in [3]). Cell type-specific markers can assist the identification but are not required for LAM. During the procedures, alterations of cellular processes are unlikely because the tissue is rapidly fixed prior to embedding, sectioning, and LAM [7]. However, only limited amounts of RNA can be isolated from rare cell types and obtaining sufficient amounts for transcriptome analysis usually requires RNA amplification. Several methods for linear RNA amplification suitable for microarray analysis have been successfully established, leading to new insights into the transcriptional state of specific cell types [1]–[6]. Nonetheless, microarrays have several limitations: high background levels due to cross-hybridization, a lack of sensitivity at low and high expression levels, and reliance upon existing knowledge about the genome sequence [8]. The recently developed high-throughput sequencing of RNA using next-generation sequencing platforms (RNA-Seq) has the potential to overcome these limitations [8], [9] and offers a variety of new possibilities such as the transcriptional profiling of organisms lacking sequence information [10], or the identification of novel loci, alternative splicing events [11], and sequence variation [12].

Given the advantages and opportunities offered by RNA-Seq, a combination of LAM and RNA-Seq promises to be a valuable tool for the transcriptional profiling of individual cell types. We expected that RNA-Seq would provide a more comprehensive view on the transcriptomes of specific cell types in means of completeness and complexity. That is, the detection of a larger number of expressed genes, the identification of transcripts from previously unannotated loci, and the description of genome-wide transcriptional patters. We therefore established the combination of LAM, linear RNA amplification, and RNA-Seq using the Life Technology SOLiD platform.

**Figure 1:**
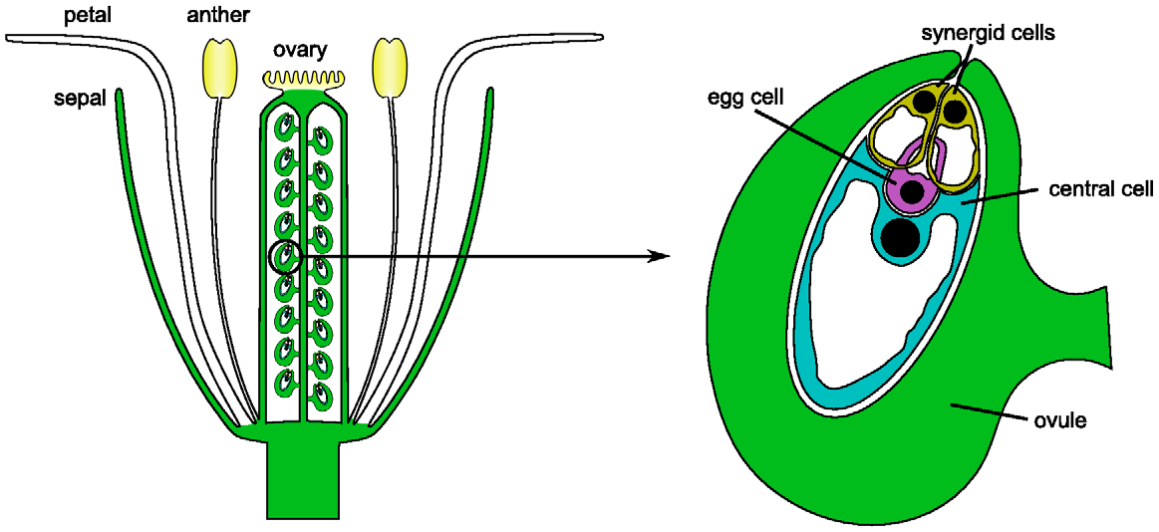
Schematic representation of the flower and the embryo sac of *Arabidopsis thaliana*. The flower of *Arabidopsis thaliana* consists of four whorls of organs: sepals, petals, anthers (male reproductive organs) and carpels (female reproductive organs). The carpels are fused and form the ovary, which harbors around fifty ovules. During ovule development, one embryo sac is formed within each ovule. The mature embryo sac contains three distinct cell types: the synergids and the two female gametes: the egg and the central cell [13]. The mature embryo sac of *Arabidopsis thaliana*, accession Landsberg *erecta*, is around 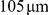 long and 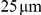 wide [44]. The nuclei of the cells of the embryo sac are drawn as black circles, the vacuoles as white regions.

As a model system we used the highly inaccessible female gametophyte (embryo sac) of *Arabidopsis thaliana* (Figure 1). The embryo sacs develop within the ovules which are themselves located within the ovary of a flower. The development of an embryo sac starts with a functional megaspore (meiotic product) that undergoes three mitotic divisions in a syncytium. Nuclear migration and concomitant cellularization eventually leads to the formation of an eight-nucleate, seven-celled female gametophyte. At maturity, the embryo sac contains three distinct cell types: the synergids and the two female gametes: the egg and the central cell [13] that, following fertilization, give rise to the embryo and endosperm, respectively. These cells are therefore good examples of rare cell types which are difficult to access. The transcriptomes of these cell types have only recently been determined using LAM in combination with Affymetrix ATH1 microarrays [3], making them an ideal system to establish the combination of LAM and RNA-Seq and to compare the two transcriptome profiling techniques.

In this study, we isolated RNA from central cells collected by LAM, prepared sequencing libraries following a protocol designed for the transcriptome analysis of a single cell [14], and sequenced them using the Life Technology SOLiD platform. We estimate the number of expressed genes (defined by having at least five reads within one replicate) to be more than twice the number reported previously in a study using LAM and ATH1 microarrays [3], and identified several intergenic regions which are likely to be transcribed. We further describe a considerable fraction of reads mapping to introns and regions close to the borders of known loci, indicating extensive alterations during transcription. Finally, we performed a *de novo* assembly of the transcriptome and showed that the workflow could also be used to study organisms lacking a reference genome. Taken together, the results indicate superior performance of the workflow presented here over the frequently used approach that combines LAM with transcriptome microarrays. We believe that the approach established in this study can be used for the cell type-specific transcriptional profiling of most eukaryotic organisms, and thus, significantly contributes to the understanding of the molecular processes underlying the development of multicellular organisms.

## Results and Discussion

### RNA isolation, library preparation and sequencing

We used LAM to dissect the central cells of the mature embryo sac. After the isolation of the cells, we used larger sections from the remaining tissue to monitor the RNA integrity with Agilent's Bioanalyzer. As a measure for this, the machine provides the RNA Integrity Number (RIN) with a range of 1 to 10, where 10 stands for a perfect RNA sample. Using an optimized version of the protocol in [3] for sample preparation, we obtained a RIN of around 8 with minor variations between different samples (data not shown).

We aimed to analyze two biological replicates (termed CC1 and CC2). Per replicate we pooled sections from approximately 450 cells during RNA extraction. Due to the low amounts of total RNA obtained with this procedure (estimated 300–1'000 pg) amplification was required. Therefore, we used the protocol described in [14], which is designed to generate cDNA libraries suitable for SOLiD sequencing from RNA isolated from a single cell. In short, mRNA is reverse transcribed to cDNA with poly(T)-primers fused to anchor sequences for PCR amplification. After PCR amplification, cDNA is sheared and amplified again after the ligation of the sequencing adapters. To monitor the efficiency of the library preparation we measured the size of the cDNAs prior to shearing using Agilent's Bioanalyzer and the approximate concentration of cDNA from selected genes with qRT-PCR (File S1). The cDNA of the first replicate (CC1) had a slightly smaller size range and lower yield than the one from the second replicate (CC2). The size distributions of around 0.1–1.5 kb indicated shortening of the RNA fragments as compared to the average full-length transcripts from *Arabidopsis thaliana* (1.5 kb). As a consequence, the sequence coverage of longer transcripts was not uniform but shifted to the 3′ ends of the transcripts (3′ bias). The bias was likely due to the oligo-dT primed cDNA generation, which has been reported to preferentially represent the 3′ ends of transcripts when compared to direct RNA fragmentation [8], [15]. However, optimized oligo-dT or direct RNA fragmentation protocols, such as described in [15], rely on mRNA enrichment and are therefore not suitable for the low amounts of total RNA obtained with the methods described here [16].

The libraries were sequenced using the SOLiD platform (version 3, Life Technology, Foster City). Each library was sequenced on one eighth of a slide resulting in a total number of 43'740'114 and 43'987'011 reads (50 bp) for the first (CC1) and the second (CC2) replicate, respectively. Potential sequencing errors were corrected using the SOLiD Accuracy Enhancement Tool (solidsoftwaretools.com/gt/project/saet). We first analyzed the data using an approach that is based on the alignment of reads to the *Arabidopsis thaliana* reference genome.

### Data analysis using a reference genome

The corrected reads were aligned to the *Arabidopsis thaliana* reference genome (www.arabidopsis.org) using TopHat [17], which is designed to identify previously undescribed splice junctions. To avoid a potential underestimation of expression levels of gene family members with similar transcript sequences we allowed up to ten alignments per read. The alignments of those reads were then weighted based on the number of uniquely aligned reads in the neighborhood. By these criteria, around 42% of the reads had at least one valid alignment, corresponding to 18'907'766 (CC1) and 18'038'960 (CC2) weighted alignments (in the following sections we use “hits” as a synonym for an alignment that was weighted).

#### Genome-wide patterns

To get a genome-wide overview of the results, we classified the hits based on their location in the genome (Table 1). The majority of the hits was found within exons and across splice junctions (82.6%). The other hits could be divided into four groups with hits (i) mapping to intronic regions (8.5%), (ii) located at or very close (distance below 200 bp) to the borders of known loci (4.8%), (iii) overlapping with known transposable elements in the intergenic regions (1%) and (iv), isolated from any known genomic feature (3.1%). For each group we then obtained the genomic regions which were sequenced in both replicates and calculated the number of hits overlapping with these “reproducibly sequenced” regions (Table 1). Overall, the sequence coverage between the replicates was highly similar with around 97.1% of all hits overlapping with a reproducibly sequenced region. Hits specific to one replicate were likely caused by a slightly differential amplification efficiency between the replicates. Furthermore, it is also possible that a higher sequencing depth would improve the similarity between the replicates in terms of sequence coverage. Nonetheless, the high percentages clearly indicate a good reproducibility of sequence coverage on a genome-wide scale.

**Table 1:** Classification of alignments.

genomic region	CC1	CC2
genome and splice junctions (total)	18'907'766.00 (97.93%)	18'038'960.00 (96.22%)
exons and splice junctions	15'456'413.54 (98.50%)	15'069'463.75 (96.65%)
introns	1'652'728.54 (93.66%)	1'485'453.60 (92.67%)
regions flanking loci	977'004.27 (95.66%)	797'708.77 (94.22%)
transposable elements outside of loci	200'268.10 (94.34%)	166'855.87 (94.39%)
remaining intergenic regions	621'351.56 (93.23%)	519'478.01 (91.56%)

The table summarizes the number of hits found in a certain genomic region. The percentage of these hits which were overlapping with regions sequenced in both replicates are given in parentheses.

**Figure 2:**
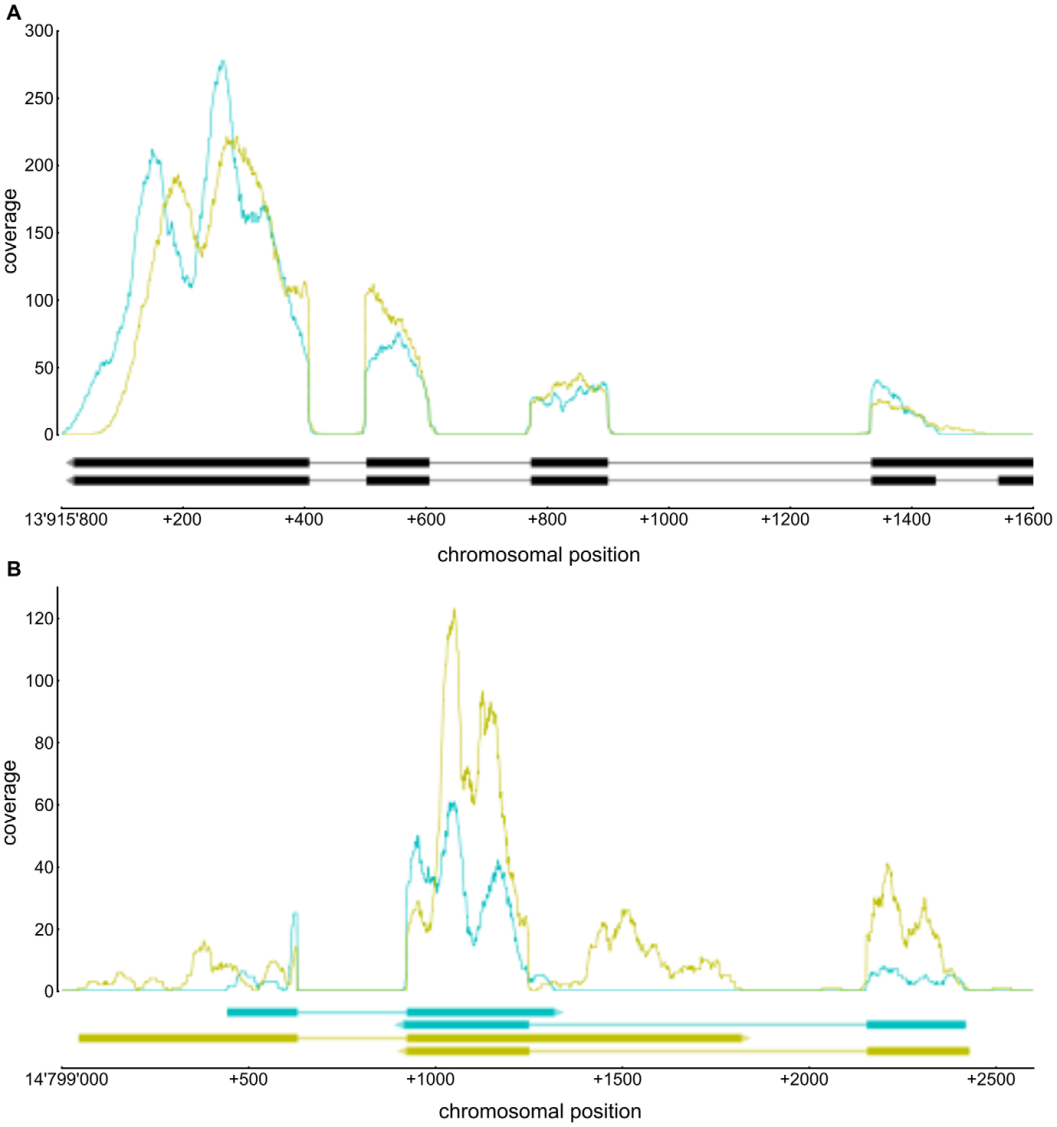
Examples of sequence coverage in annotated (A) and unannotated (B) regions. Graphs in the upper parts of the panels represent the number of hits per base within the two replicates (CC1: cyan, CC2: yellow). Transcripts are drawn in the lower parts of the panels: dark boxes represent exons, bright lines mark introns and the arrowhead depicts the direction of transcription. (A) Sequence coverage at the region around the locus *AT4G27960* (*UBC9*) on chromosome 4. The two transcripts represent two isoforms of *AT4G27960*. Clearly visible is the lack of coverage at the introns and the non-uniformity of sequence coverage with the maxima close to the 3′ end of the transcripts. (B) Sequence coverage at a region on chromosome 5, which is not annotated as being transcribed. Hits in this region were assembled into transcripts using cufflinks [11]. For each replicate, two transcripts with overlapping 3′ ends could be assembled (CC1: cyan, CC2: yellow). Notably, the sequence coverage along these transcripts resembles the coverage observed at annotated transcripts (A). Also visible are the unsharp transcript boundaries which vary between the replicates.

Overall, the percentage of non-exonic hits (in total 17.4% of all hits) is well above the percentages reported in other RNA-Seq studies on *Arabidopsis thaliana* (pool of organs and seedlings in [18]: 7%; unopened flower buds in [19]: 3.5%). An explanation for this difference may be that the annotation of the *Arabidopsis thaliana* genome is widely based on sequencing of cDNAs and expressed sequence tags (ESTs) originating from larger plant structures or whole plants. Transcripts uniquely expressed in small structures or rare cell types, such as the female gametophytic cells, were therefore less likely to be detected due to a dilution effect. Thus, the high percentage of intergenic hits in the data presented here may partly reflect transcripts or transcript isoforms specific to the central cell. Detailed analysis of transcript isoforms from known loci is, however, difficult due to the non-uniform sequence coverage. Nonetheless, we used the intergenic hits which were isolated from any known genomic feature to search for (fragments) of transcripts from previously unannotated loci. To identify these loci we used cufflinks [11], which is designed to assemble transcripts from reads which were aligned to a reference genome (with the focus on paired-end read libraries). Using single-end and unstranded reads, the program assumes uniform coverage along a transcript. It is therefore not well suited for an in-depth analysis of data generated with the methods presented here. Nonetheless, we could identify 78 (CC1) and 115 (CC2) potentially new transcripts, which were supported by one or more splice junctions (Table S1). Many of them showed a coverage pattern similar to the one observed for annotated transcripts (example in Figure 2B). These transcripts corresponded to 75 (CC1) and 112 (CC2) putative loci, in the following termed “splice-loci”. To compare their genomic location between the two replicates, we calculated for each of them the overlap with a splice-locus/loci from the other replicate and counted the number of splice-loci with an overlap above a certain threshold (Figure S1). Splice-loci with very low expression values (less than 25 hits) showed a poor overlap between the two replicates, irrespective of the threshold (11% with perfect overlap and 16% with an overlap of at least 10%). Reasons for this may be a higher variability between the two replicates at low expression levels, a stronger influence of sequencing or alignment errors, and a sparse read coverage leading to a highly fragmented assembly. Splice-loci with higher expression values exhibited substantially higher overlaps, ranging from 17% (perfect overlap) to 48% (overlap of at least 10%). However, the number of splice-loci with an overlap above a certain threshold increased substantially, when overlaps of splice-loci with loci from transcripts not supported by splice junctions were also considered to be valid (19% perfect overlap, 85% with an overlap of at least 10%), likely indicating a fragmented assembly due to a lack of gapped alignments. Given that the assembly, especially of transcripts with low to moderate expression levels, is strongly depending on sequencing depth [11], we expect that an increased sequencing depth together with the use of paired-end reads would improve the assembly and thus, the overlap between the replicates. Taken together, we suggest that the potentially new transcripts identified in this study with cufflinks should be considered as preliminary, still requiring further experimental exploration and validation. Nonetheless, we consider cufflinks as a valuable tool to start the search for potentially new transcripts in unannotated regions. It provides a basis to explore so far unknown transcribed regions also by other methods such as sequence alignment or gene prediction.

#### Transcriptional profiling

**Figure 3:**
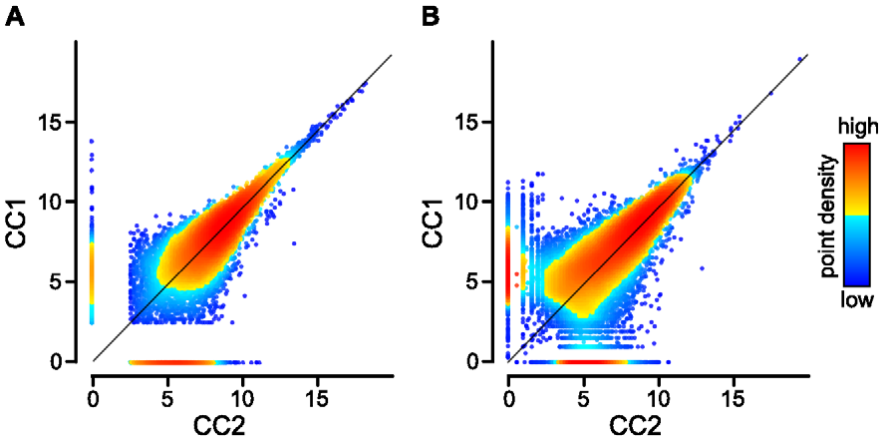
Comparisons of expression values between the two RNA-Seq replicates. In each panel, the expression values (log2 of the number of hits plus one) for each feature are plotted on the x-axis (CC2) and the y-axis (CC1). Colors indicate the point density: red and blue indicate the highest, respectively lowest, densities. (A) refers to the approach that was based on the alignment of reads to the reference genome: given are the expression values of the “expressed” genes (Pearson correlation: 0.99, Spearman correlation: 0.83). (B) refers to the approach that was based on *de novo* assembly of the short reads. Reads from both replicates were pooled and assembled together. To calculate expression values, reads from both replicates were aligned to the assembled transcriptome (Spearman correlation: 0.87).

To get an overview of the hits mapping to annotated transcripts, we visualized the coverage at the transcripts (example in Figure 2A). This confirmed a 3′ bias, which was likely introduced during cDNA synthesis, within the data [15]. The 3′ bias partly counteracts the transcription length bias discussed in [20], due to a non-uniform coverage along a transcript. The relationship between the number of hits per transcript and its length is therefore only linear at the 3′ end of the transcript where the coverage is still uniform. Assuming a linear relationship over the entire length would thus lead to an underestimation of expression values from longer transcripts (e.g. RPKMs in the ERANGE software [21]). A possibility would be to take only hits in a certain distance to the 3′ end. However, this would exclude a certain proportion of the data [20]. We therefore decided to use the total number of hits mapping to the transcripts of a locus as expression value for the locus. Hits mapping to more than one locus (ambiguous hits) were proportionally distributed based on the number of unambiguous hits. Loci with transcripts having less than five hits or no hit located within the 250 bps at the 3′ end were discarded, the others declared as being expressed. Of the 33'598 annotated genes, pseudogenes, and transposable element genes, 17'419 (51.8%) met these criteria in at least one of the replicates (Table S2). Among these genes, 13'426 were found within both replicates. The other 3'993 loci were specific to one of the replicates (CC1: 1'028, CC2: 2'965). These loci had generally low expression values in the replicate in which they were detected (Figure 3A). It is therefore possible that a higher sequencing depth would reduce the number of replicate-specific loci. Beside this difference within the presumably low abundant transcripts, the replicates were highly similar (Figure 3A). However, the differences highlight the importance of replication that is necessary to estimate the variability in the data, especially of the genes with presumably low expression levels.

To compare the data generated with RNA-Seq to the one measured with the ATH1 microarrays [3], the expression values of the RNA-Seq data were transformed (log2(x+1)). ATH1 expression values and present calls were obtained as described in [22] (Table S2).

**Figure 4:**
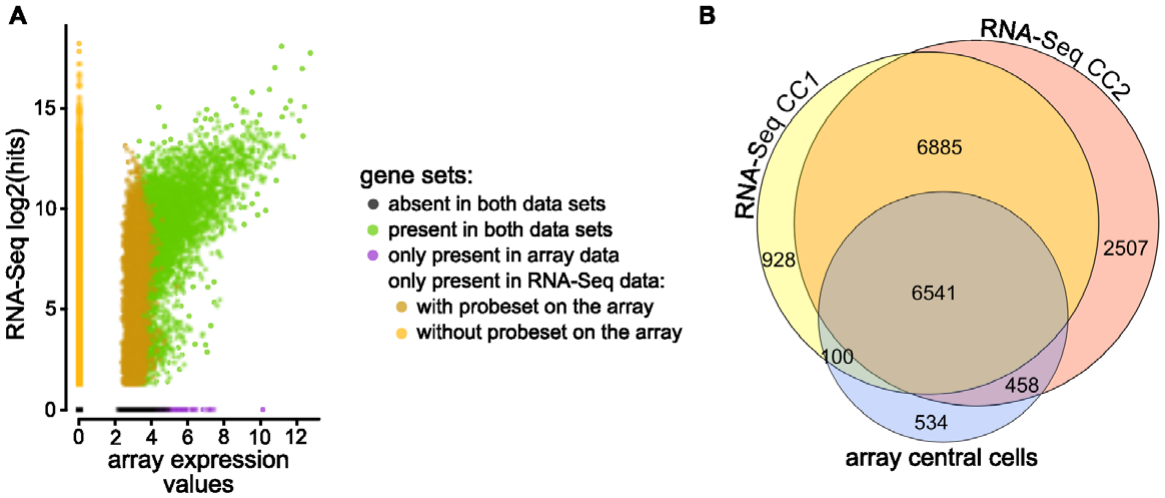
Comparisons between microarray and RNA-Seq data. (A) The average number of hits (log2(x+1)) for each gene are plotted on the y-axis and the corresponding normalized expression values from the array data are shown on the x-axis. Expression values of the genes having a probeset on the array are well correlated between the technologies (Spearman correlation: 0.63). (B) A Venn diagram summarizing the overlap between genes detected to be expressed in the RNA-Seq data sets and the array data.

We first compared the average expression values. Using only the genes which have a corresponding probe set on the ATH1 array (21'440), we found that the measures of transcript abundance were well correlated (Figure 4A). Differences could be observed where array expression values were relatively high and the expression values from the RNA-Seq data small or zero (in agreement to [9]). This observation may be due to probe-specific background hybridization on the array [9].

We next compared the transcriptomes. From the 7'633 genes which were found to be expressed in the ATH1 array data, 93% were also detected in the RNA-Seq data (Figure 4B). The remaining 7%, exhibited medium expression values in the array data (Figure 4A). As mentioned before, it is possible that expression values for some of those genes were elevated due to probe-specific background hybridization. In addition to the 7'099 genes found in both data sets, 10'320 genes were only detected in the RNA-Seq data. From these, 34.6% were *a priori* not measurable using the ATH1 array because it lacks the corresponding probesets. The other 65.4% had low expression values in the array data. It is therefore likely that these signals could not be separated from the background [23].

#### Comparing RNA-Seq and ATH1 array data from central cells

**Figure 5:**
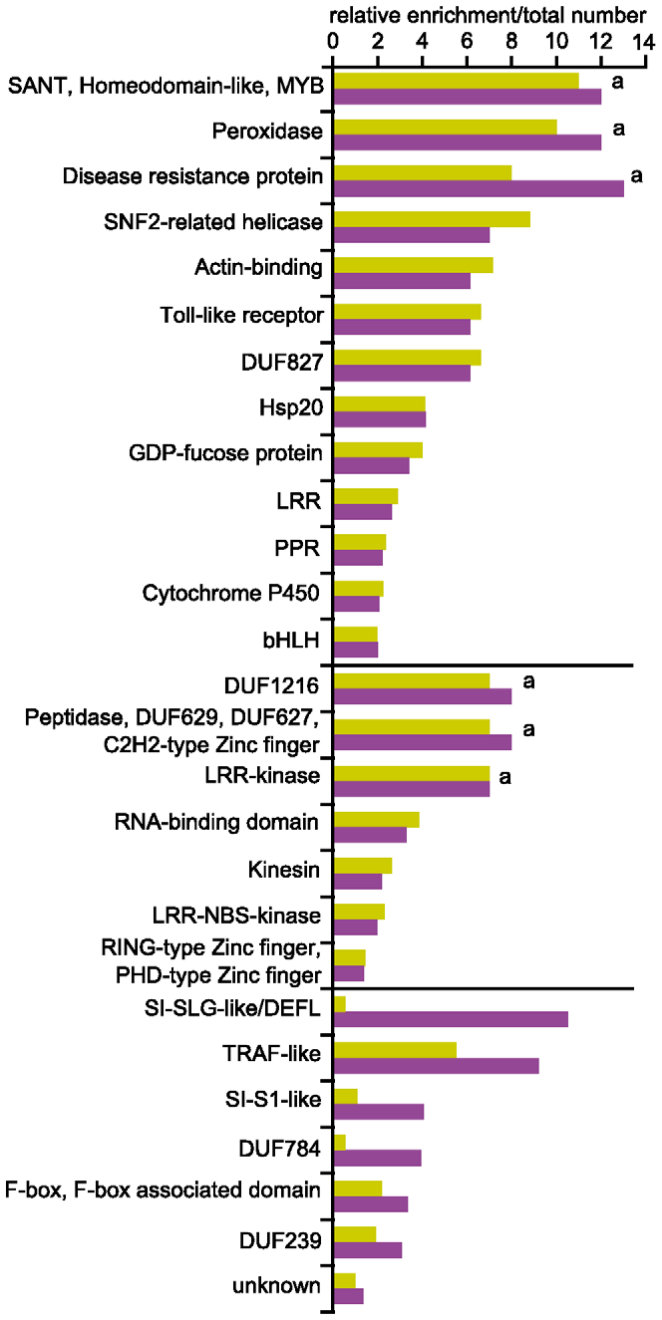
Test for enrichment of InterPro domains in RNA-Seq data compared to array data. The graph shows the relative enrichment of (combinations of) InterPro domains (simplified description, details are given in Table S3) in the RNA-Seq data compared to the array data, which was found to be significant. If the combination did not occur in the array data, the enrichment value was set to the total number of occurences of the combination in the RNA-Seq data (marked with a). We performed two tests to separate the effect of the higher sensitivity (yellow) from the effect caused by the whole-genome coverage (magenta). Combinations of protein domains in the upper, middle, and lower part of the figure were significantly enriched in both, the first, and the second test, respectively. Abbreviations: DUF: domain of unknown function, LRR: leucine rich repeat, PPR: pentatricopeptide repeat, bHLH: basic helix-loop-helix, NBS: nucleotide binding site, SI-: self-incompatibility, DEFL: defensin-like. The term “unknown” comprises all transcripts without an InterPro annotation (includes also non-protein-coding genes).

Given the differences in the size of the transcriptomes, we investigated a potential effect on the functional characterization of the central cell transcriptome. In terms of gene functions, the transcriptome measured with microarrays may either be a representative subset of the transcriptome determined using RNA-Seq or show a systematical over- or underrepresentation of genes having certain functions. Considering that the few array-specific genes were likely to be false positives, such systematic overrepresentation of functional groups in the array data would arise either from those false positives or from a sampling artifact, which may occur if a certain functional group was very well characterized at the time the ATH1 microarray was designed, and thus, almost entirely represented on the array. We therefore only tested for overrepresentation of transcripts encoding a given combination of protein domains (InterPro, www.ebi.ac.uk/interpro) in the RNA-Seq data compared to the array data (Figure 5, Table S3). Enrichment in the RNA-Seq data may originate from specific groups of genes newly detected due to either the higher sensitivity, which increases the propability to detect low expressed genes, or the whole-genome coverage that allows to detect genes which are not measurable with the ATH1 microarrays due to a lack of the corresponding probeset. The latter is a consequence of the ATH1 microarray design and would not occur with arrays covering the whole genome. We therefore performed two tests to separate the two effects from each other.

To test for enrichment likely caused by a higher sensitivity, we compared the functional characterizations of the transcriptomes determined using the array or the RNA-Seq data and excluded the genes missing a corresponding probeset on the ATH1 microarray. From 4'657 distinct (combinations of) protein domains found in this set of genes, 20 were significantly enriched in the RNA-Seq data compared to the array data (Fisher's exact test, one-sided p-value<0.05). Among them, (combinations of) domains which might play important roles in cell fate determination were identified: signal perception and transduction (Toll-like receptor), chromatin remodeling (SNF2-related helicase), regulation of transcription (SANT, Homeodomain-like, MYB), and RNA-binding (Figure 5).

To estimate the effect of the whole-genome coverage on the functional characterization, we performed a second enrichment analysis, which included also the genes missing a corresponding probeset on the ATH1 microarray and could identify seven additional (combinations of) protein domains showing significant enrichment in the RNA-Seq data. The largest group comprised genes with an “unknown” domain, which included uncharacterized protein-coding as well as non-protein-coding genes. The enrichment was therefore likely due to the non-protein-coding genes from which only few are represented on the ATH1 microarray. The other six groups contained genes encoding for domains of unknown function (DUF784, DUF239), meprin and tumour necrosis factor receptor associated factor homology domains (TRAF-like), F-box and F-box associated domains, S1 self-incompatibility related proteins (SI-S1-like), and small cysteine rich defensin-like proteins (SI-SLG-like/DEFL, Figure 5). Interesting to note is that the latter were implicated as signaling molecules required for pollen tube guidance in *Zea maize* and *Torenia fournieri* [24], [25]. In *Arabidopsis thaliana* they might contribute to the role of the central cell in pollen tube guidance [3], [26] or, what remains to be examined, as well function as signaling molecules within the mature embryo sac.

Taken together, we found that the two technologies correlate relatively well. Most of the transcripts detected in the array data were also detected in the RNA-Seq data (7'099). However, we could identify additional 10320 genes which are likely to be expressed in the central cell. A third of those could not be measured with the ATH1 microarray due to the lack of the corresponding probesets. This differences are therefore largely due to the ATH1 microarray design and would not occur in experiments using microarrays with whole-genome coverage. Importantly, however, the other two thirds could be attributed to the higher sensitivity of RNA-Seq compared to microarrays. Interestingly, this did not only increase the estimated size of the transcriptome, but also had an effect on the functional characterization of the transcriptome. Given that RNA-Seq is highly accurate [8], [9], [21], [27], the results demonstrate the superior performance of RNA-Seq over the array based method for the transcriptional profiling of specific cell types. Nonetheless, at the moment microarrays still have certain advantages. Numerous tools were developed, tested, and used extensively for analysis of data from a broad range of experiments, offering reliable and efficient data analysis for almost any experiment. Compared to this, RNA-Seq data analysis is still a relatively new field of research which, however, evolves rapidly. Experience with the available tools is therefore rather limited compared to the ones used for microarray data analysis. Another advantage concerning the microarrays, which are frequently used, is the vast amount of publicly available data sets generated over the past years. For *Arabidopsis thaliana*, data from more than 7000 ATH1 microarrays are currently available on NCBI (www.ncbi.nlm.nih.gov). This offers the possibility to relate a newly determined transcriptome to many others, as for example presented in [3] where the transcriptomes of the cells from the female gametophyte could be directly compared to the ones of 59 different tissues or cell types. However, these advantages are likely to decrease fast as it is most probably only a matter of time until RNA-Seq will be the method of choice for transcriptional profiling [28].

#### Genes specifically expressed in central cells

A frequent application of transcriptional profiling is the analysis of differential expression of genes between different tissues and cell types or time points. With this approach, Wuest and coworkers [3] could identify 431 genes (FDR<0.05) which are likely to be specifically expressed in the mature female gametophyte as compared to 59 different tissues and cell types from *Arabidopsis thaliana*. Most of them were specific to one of the three cell types (113, 163, and 144 in the central cell, egg, and synergid cells, respectively). Functional characterization further revealed an enrichment of specific posttranscriptional regulatory modules and metabolic pathways in each cell type [3]. Given the higher sensitivity of RNA-Seq and the whole-genome coverage, we expect that an analysis using transcriptomes measured with RNA-Seq would provide an even deeper insight to the unique nature of the transcriptome of the mature female gametophyte. However, the small number of publicly available RNA-Seq data from *Arabidopsis thaliana* and the lack of RNA-Seq data from egg and synergid cells hamper an in-depth analysis as performed in [3]. Nonetheless, to get preliminary insights into the unique nature of the central cell transcriptome, we performed a comparison of the central cell transcriptome with transcriptomes from other tissues and cell types from *Arabidopsis thaliana*, which had been analyzed by RNA-Seq. The test set comprised data from 2–4 cell and globular stage embryos [12], early globular embryos [29], whole plants (pool of organs) [18], seedlings [18], unopened flower buds [19], and male meiocytes [30], thus representing a diverse set of tissues and cell types.

**Figure 6:**
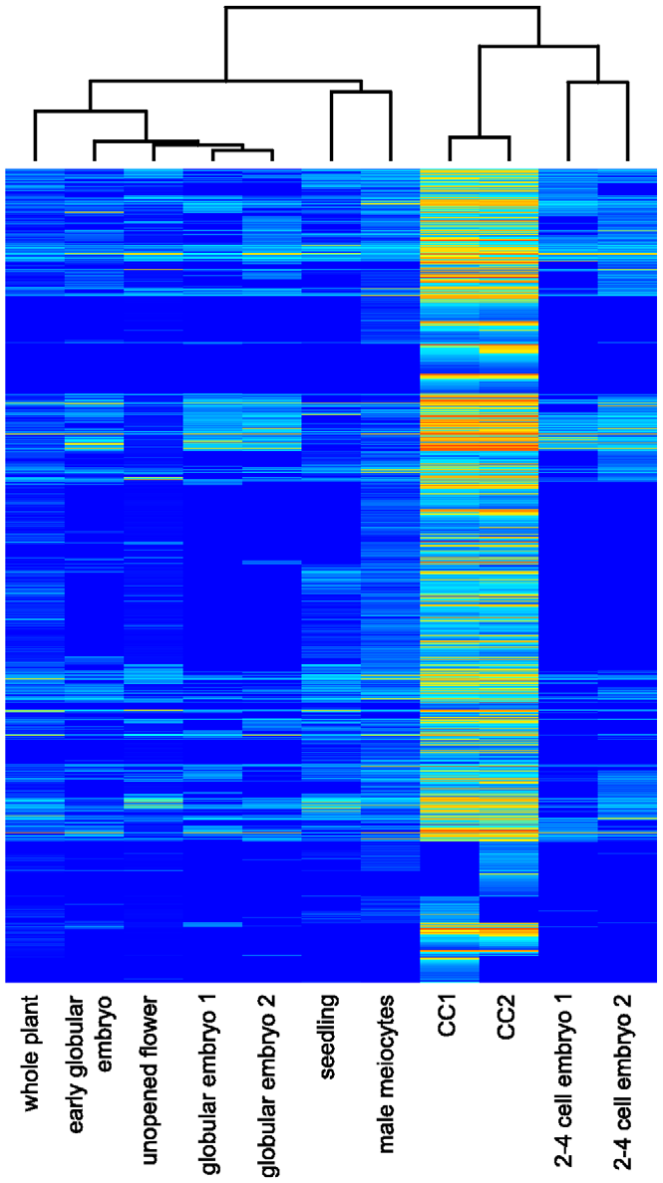
Genes enriched in the central cell compared to other tissues of *Arabidopsis thaliana*. Expression values of genes preferentially expressed in central cells are summarized in a heatmap (blue/red: low/high expression values). Expression values were equalized using edgeR [31] and log2(x+1) transformed. Samples and genes were clustered using Spearman correlation and hierarchical agglomerative clustering. Transcriptomes from whole plant and seedlings, unopened flowers, early globular embryos, male meiocytes, and 2–4 cell and globular stage embryos were obtained from [18], [19], [29], [30], and [12], respectively.

Using edgeR [31] to test for differential expression, we could identify 1'418 genes (FDR<0.05) upregulated in the central cell compared to the other tissues and cell types (Figure 6). We could thereby confirm 75% of the genes previously found to be specific to the central cell [3]. We also found that 9% and 17% of the genes previously described as enriched in the egg and the synergid cells, respectively, showed significant enrichment in the central cell in our comparison. Cross-contamination is however unlikely considering that the central cell is very well separatable from the egg and the synergids. In addition, one would rather expect contamination from the egg cell instead of the synergids, as the egg is closer to the central cell than the synergids. We therefore suggest that the difference was likely an artifact due to the lack of RNA-Seq data from the egg and synergid cells: In our comparison, genes expressed in central cells at a level below the one in egg or synergid cells but above the level in all other tissues were identified as specifically enriched. However, if data from egg and synergid cells were included, these genes would not be identified as being enriched in central cells.

To characterize the set of genes found to be specifically enriched in the central cell, we used the InterPro annotation (www.ebi.ac.uk/interpro) and tested for enrichment of certain (combinations of) protein domains as described above (Table S4). We found 118 and 11 (combinations of) protein domains showing enrichment in the central cell at a low stringency (p-value<0.05) and high stringency (FDR<0.05) cutoff, respectively. Among the most significantly enriched, are several domains that are underrepresented on the ATH1 array: domains of unknown function (DUF784, DUF239), F-box and F-box associated domains, S1 self-incompatibility related proteins, and small cysteine rich defensin-like proteins (DEFLs). These results indicate that genes specific to the mature female gametophyte are generally underrepresented on the ATH1 array as observed previously [32]. However, even though underrepresented on the ATH1 array, several of them (F-box, DUFs, DEFLs) were already identified previously to be specifically enriched in the mature female gametophyte [3], with the DEFL being highly specific to the central cell, thus indicating good agreement between the comparisons performed in this study and [3]. A similar overlap could also be observed for several (combinations of) protein domains which may play an important role in cell fate determination, such as the type I MADS-box and RWP-RK transcription factors. Examples for functional groups not identified in [3] comprise several genes encoding for diverse epigenetic functions including a histone methyltransferase (*AT2G24740/SUVH8*), a chromomethylase (*AT1G80740/CMT1*), and two DNA-methyltransferases (*AT4G08990* and *AT4G14140/MET2*), which may contribute to the specific epigenetic state of the central cell [33], [34].

Taken together, the enrichment analysis presented in this study widely agrees with previously reported results [3] in terms of functional enrichment, but extends the number of specific genes to a large extent. However, given the few RNA-Seq data sets in the comparison and the lack of the egg and synergid transcriptomes, these results are preliminary, requiring additional data sets for the comparison in order to obtain a more thorough view on the unique nature of the transcriptome of the central cell.

### Data analysis using *de novo* transcriptome assembly

RNA-Seq offers the possibility to investigate an organism which lacks sequence information. To test whether the methods presented here are suitable for such a study, we performed a *de novo* assembly of short reads and briefly characterized the transcriptome using GO terms. An in-depth analysis of the results is, however, out of scope of this article.

*De novo* assembly of RNA-Seq data into transcripts is generally challenging due to the non-uniform sequencing coverage across transcripts, the presence of low quality reads, and the size of the data sets [35]. In the data presented here, additional complexity is caused by fragment shortening introduced during library preparation, which leads to a non-uniform sequence coverage within the individual transcripts. To overcome some of the limitations, we first corrected potential sequencing errors and then removed all reads which were of low quality, repetitive or duplicated. The remaining reads were assembled using velvet [36], oases, and additional tools, which were required to handle the color space of SOLiD reads. Expression values were obtained using the full set of reads and bowtie [37]. To characterize the assembled transcripts, we used Blast2GO, which is designed to annotate (protein coding) sequences based on similarity searches and existing annotation associations [38]. Transcripts were first mapped against NCBI's non-redundant protein sequence database (www.ncbi.nlm.nih.gov) using BLAST [39] with an e-value threshold of 1e-6 and a maximum of 20 blast hits. Gene Ontology (GO) terms [40] were obtained and assigned using default settings.

From the initial set of reads containing reads from both replicates, around half (44'686'342) passed the filter criteria. From these, around 28.7% were used during the assembly, resulting in 32'567 transcripts with an average length of 307.1 bp (File S2) and a sequence coverage resembling the results from the previous analysis; beside the differences for replicate-specific transcripts, sequence coverage was highly similar (Figure 3B). From the 32'567 assembled transcripts, 19'502 had one or more blast hits to the non-redundant protein sequence database. Most (89.4%) of them had the best blast hit to *Arabidopsis thaliana* or its close relative *Arabidopsis lyrata*. In the latter case, the transcripts generally also mapped to *Arabidopsis thaliana* sequences. The majority of the remaining best blast hits were found among fungal pathogens affecting plants (3.8%) and animals (1.8%). Transcripts with hits to fungal species originated from the first replicate (CC1) and were not found in the second replicate (CC2), indicating some contamination of the RNA from CC1 (replicate-specific assemblies, data not shown). Interestingly, reads aligning to those sequences do generally not align to the genome of *Arabidopsis thaliana* (0.0016% of all reads aligning to the genome do align to the sequences of those fungi). It is therefore unlikely that the contamination affected the alignment-based approach described before.

To compare the assembled transcriptome to the transcriptome determined in the alignment based approach, the two transcriptomes were annotated and compared at the level of GO terms using Blast2GO [38]. To avoid a bias due to the fungal contamination, we only used the assembled transcripts with the best blast hit to either *Arabidopsis thaliana* or *Arabidopsis lyrata* for the comparison. From these 17'641 transcripts, 14'514 could be annotated with 4'859 distinct GO terms. Overall, the number of distinct GO terms per annotated transcript was lower in the transcriptome from the *de novo* assembly compared to the transcriptome determined with the alignment based approach described before, where 14'487 of the 17'419 transcripts could be annotated with 5'285 distinct GO terms (only one, the representative, transcript per locus). However, the distribution of GO terms belonging to the domain “Molecular Function” showed a high similarity between the two transcriptomes: only 10 terms showed significant enrichment in one of the transcriptomes (two-sided Fisher's exact test, FDR<0.05). The most specific terms among them were: structural constituent of ribosome (GO:0003735), transcription factor activity (GO:0003700), RNA binding (GO:0003723), protein serine/threonine kinase activity (GO:0004674), and translation factor activity/nucleic acid binding (GO:0008135). All those terms displayed an enrichment in the assembled transcriptome. For genes related to transcription factor or protein kinase activity this was unexpected as they are often expressed at low levels. However, it is possible that the marginal coverage of these transcripts caused a fragmented assembly: if the reads from one transcript were assembled into two fragments of the transcript, from which both map to the GO term, the GO term would be counted twice, thus leading to an overrepresentation of the respective GO term.

Taken together, the results of this test indicate that data obtained with the methods presented here is in principle suitable for *de novo* assembly of a transcriptome: sequence coverage patterns and GO annotations largely resembled the ones found in the alignment-based approach. However, it is likely that many of the assembled transcripts were shorter than *in vivo* due to the 3′ bias. In cases where most of the assembled sequence contained mainly untranslated regions (long 3′ UTR), it probably had an effect on the GO term annotation (which is based on similarity to known proteins). Considering further that the annotation using GO terms can only characterize protein-coding transcripts, we recommend to use additional methods for the annotation and analysis of the assembled transcripts. One possibility would be to search databases containing all types of transcripts for similarity in the nucleotide sequence. We expect that this would help to characterize non-coding transcripts and improve the GO annotation of protein-coding transcripts which could not be annotated using the methods relying on similarity to proteins.

### Conclusion

We aimed to establish a workflow that allows determining the transcriptional profile with a high sensitivity and resolution of specific cell types, which are very rare and difficult to access as they are embedded in heterogenic tissue. We therefore combined LAM with a highly sensitive, linear RNA amplification method and the emerging RNA-Seq technology. As a model we used central cells of *Arabidopsis thaliana* from which only around 50 are formed within a flower, each of them individually enclosed by an ovule. Using LAM, we could obtain sufficient amounts of good quality RNA for a successful amplification and library preparation. We compared the data generated in this study with the transcriptome data from [3], which was measured using LAM and the ATH1 microarray. The results showed that the two transcriptome profiling technologies correlate well. Most of the genes found to be expressed in the microarray data were also present in the RNA-Seq data and the few microarray specific genes were likely false positives caused by probe specific cross-hybridization. However, using RNA-Seq we could detect more than double the amount of presumably expressed genes. Functionally, this difference was reflected in the enrichment of genes encoding for few specific (combinations of) protein domains, of which some may play an important role in cell fate determination (signal perception and transduction, chromatin remodeling, and regulation of transcription) or function of the specific cell type (defensin-like proteins), in the RNA-Seq data compared to the array data. In addition, we identified several intergenic regions which are likely to be transcribed. We further described a considerable fraction of reads aligning to introns and regions flanking annotated loci which may represent alternative transcript isoforms. Finally, we also performed a *de novo* assembly of short reads and briefly characterized the assembled transcriptome. Comparisons between the alignment- and the assembly-based approaches revealed that the results were remarkably similar in terms of sequence coverage pattern and Gene Ontology (GO) annotation, indicating that the workflow presented here is also suitable to study specific cell types from an organism lacking a reference sequence. Taken together, we successfully established an easy and reliable workflow that allows the transcriptional profiling of specific cell types, which are rare and difficult to access, with high sensitivity and resolution. The approach presented here will provide new insights into the transcriptional state of individual cell types not only of plants, but also other eukaryotes and, therefore, by elucidating cell fate decisions, will contribute to the understanding of the molecular processes underlying the development of multicellular organisms.

## Materials and Methods

### Plant material

*Arabidopsis thaliana* seeds, accession Landsberg *erecta*, were germinated on Murashige and Skoog agar (0.5× Murashige and Skoog salts, 0.7% phytagar). One week old seedlings were transferred to ED73 soil (Einheitserde, Schopfheim, Germany), and grown under 16 h light at 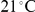 and 8 h darkness at 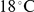 and 60% relative humidity.

### Tissue embedding

Two days after emasculation, flowers containing the mature embryo sacs were fixed in ethanol∶acetic acid 3∶1. Vacuum was applied two times for 15 min at 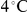. Afterwards the material was kept in the fixative overnight at 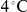 and subsequently transferred to an ASP200 embedding machine (Leica Microsystems GmbH, Wetzlar, Germany). In the embedding machine, tissues were dehydrated automatically in a graded series of ethanol (70% for 1 h, 3×90% for 1 h, 3×99.98% for 1 h, all at room temperature) followed by xylol (2×1 h and 1×1 h 15 min, all at room temperature). Xylol was substituted by Paraplast X-tra embedding media (Roth AG, Arlesheim, Switzerland) at 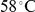 (2×1 h and 1×3 h). Finally, flowers were poured into paraffin blocks, cooled, and stored at 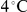.

### Laser-assisted microdissection

For microdissection, paraffin blocks containing flowers were cut on a RM2145 Leica microtome (Leica Microsystems GmbH, Wetzlar, Germany) to 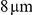 thin sections and mounted on nuclease-free membrane-mounted metal-frame slides using pure methanol ([3] used water). Slides were dried overnight on a heating table at 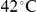. Samples were deparaffinized in xylol at room temperature (2×10 min). Microdissection was performed using a mmi CellCut Plus device (MMI Molecular Machines & Industries AG, Glattbrugg, Switzerland). Isolated central cells were collected using MMI isolation caps and stored at 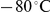. Four to six cuts of whole flowers were taken from each slide after collecting the cells of interest. Total RNA was isolated and tested for integrity using the Agilent 2100 Bioanalyzer together with the RNA 6000 Pico Kit (Agilent Technologies Schweiz AG, Basel, Switzerland).

### RNA isolation

Total RNA was isolated using the PicoPure RNA isolation kit (Arcturus Engineering, Mountain View, USA) following the manufacturer's instructions with slight modification. Caps were covered with 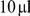 extraction buffer and incubated at 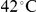 for 30 minutes. Extracts from different caps were pooled to reach a sufficient RNA yield. All other steps were performed according to the manufacturer's instructions, including the on-column DNase treatment using the RNase-free DNase set from QIAGEN (Valencia, USA).

### RNA sequencing

The amplification and library preparation of RNA from central cell samples were performed following the protocol described in [14]. Libraries were sequenced using the SOLiD platform, version 3 (Life Technology, Foster City, USA). For each library one eighth of a slide was used.

### qRT-PCR

To monitor the efficiency of the library preparation we measured the size of the cDNAs prior to shearing using Agilent's 2100 Bioanalyzer following the instructions from the manufacturer. We also estimated the concentration of cDNA from selected genes with qRT-PCR: *ACT2* (*AT3G18780*, forward: CTTGCACCAAGCAGCATGAA, reverse: CCGATCCAGACACTGTACTTCCTT, [41]), *ACT11* (*AT3G12110*, forward: AAGCTGTTCTTTCCCTCTACGC, reverse: GGAACAGTGTGACTCACACCATC, [42]), 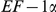 (*AT5G60390*, forward: TGAGCACGCTCTTCTTGCTTTCA, reverse: GGTGGTGGCATCCATCTTGTTACA, [43]) and *UBC9* (*AT4G27960*, forward: TCACAATTTCCAAGGTGCTGC, reverse: TCATCTGGGTTTGGATCCGT, [41]). All qRT-PCR reactions were performed in a final volume of 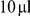 containing 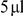 cDNA or water, 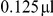 of each primer (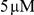), 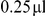 water and 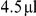 mastermix (Power SYBR Green PCR Master Mix, Life Technology) on the 7900 HT Fast Real Time PCR System (Life Technology) with the following cycling conditions: 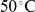 for 2 minutes, 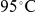 for 10 minutes and 45 times 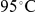 for 15 seconds followed by 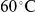 for 1 minute. Amplicon length was determined using the melting curve analysis.

### Data processing: reference genome

Short reads and alignments generated in this study were deposited at NCBI Gene Expression Omnibus (www.ncbi.nlm.nih.gov/geo) and are accessible through GEO series accession number GSE29719. Reads (csfasta and qual files) were processed with the SOLiD Accuracy Enhancement Tool (version 2.2 with a reflength of 13'000'000 and the option -qvupdate; solidsoftwaretools.com/gt/project/saet [note added in proof: SAET was moved to solidsoftwaretools.com/gf/project/denovo/frs]) and aligned to the reference genome (www.arabidopsis.org) using TopHat with the options –color –quals –coverage-search -a 8 -m 1 -i 50 -I 2000 -F 0.2 -p 7 -g 10 (version 1.2; [11]). We allowed up to ten alignments per read to avoid a potential underestimation of expression values of transcripts with similar sequence. However, a read 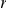 with 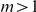 alignments would count 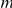 times, resulting in overestimation of expression values. To avoid this, we calculated for each alignment 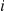 of such a read the weight 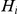 using a “score” 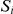 divided by the sum of scores from all alignments of the read (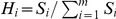). If the total score was zero, all alignments were discarded. For ungapped alignments, the score was equal to the sum of coverage originating from uniquely aligned reads at the position of the alignment and the surrounding 100 bps (“allocation distance” of 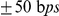). For gapped alignments, the score was equal to the number of uniquely aligned reads spanning the same gap. Thus, if a read had both types of alignments, the ungapped ones would have been preferred. Here we use “hit” as a synonym for an alignment that has been weighted.

#### Identification of new transcripts

To find potentially new transcripts in intergenic regions, we extracted all alignments that were not overlapping with a known transposable element and at least 200 bps outside of a known locus. The “intergenic” transcriptome was then assembled using these intergenic alignments and cufflinks (version 0.9.3) with a maximal intron length of 2000 [11]. To compare the genomic location of the loci from the potentially new transcripts between the two replicates, we calculated for each locus from each replicate the overlap with a locus/loci (with and without the remaining loci with transcripts not supported by splice junctions) from the other replicate (number of shared nucleotides divided by the length of the locus) and counted the number of loci with an overlap above a certain threshold. Potentially new transcripts supported by at least one splice junction were annotated using Blast2GO (version 2.4.8; [38]).

#### Transcriptome data

Hits were assigned to the transcripts of the genomic features “gene”, “pseudogene” and “transposable element gene” (TAIR10, www.arabidopsis.org). Hits can be divided into unambiguous (mapping to transcripts of only one locus) and ambiguous (mapping to transcripts of more than one locus). To avoid counting ambiguous hits multiple times, we proportionally distributed them based on the number of unambiguous hits. If there were no unambiguous hits, the ambiguous hits were equally distributed. However, we assume a case where two loci A and B overlap such that locus A is entirely located within locus B. Locus A shall be “truly” expressed, locus B not. Using a single step, all hits of locus A would be declared as ambiguous. In case locus B has no unambiguous hit, the hits from locus A would be equally distributed to locus A and B, leading to an underestimation of the expression value from locus A and an overestimation of the expression value from locus B (a false positive). In another case where locus B has one or two unambiguous hits due to sequencing and/or alignment errors, all the hits from locus A would be wrongly assigned to locus B (one false positive and one false negative). The same error would occur if locus A has a longer transcript than the annotation would indicate. The hits at the borders of locus A would then be unambiguously assigned to locus B and as a consequence also all the ambiguous hits. To avoid this scenario at least to some extent we used a two step approach. In the first step, all hits were mapped to all annotated transcripts. We expected that each “truly” expressed transcript should have at least one hit within the 250 bps at its 3′ end because the library preparation protocol was based on poly(A)-tail priming for cDNA synthesis and adapters for the amplification. In addition, we set a threshold of five hits as a minimal expression value to overcome possible sequencing and alignment errors. Transcripts not matching these criteria were discarded. During the second step, the hits were divided into unambiguous and ambiguous. The unambiguous hits were assigned first and used to distribute the ambiguous hits. The transcripts were then filtered again using the same criteria as before. The final expression value of a locus was calculated as the sum of hits assigned to any of its transcripts. Expression values are given in Table S2.

#### Enrichment of combinations of protein domains (InterPro)

Genes present in array data (i), RNA-Seq data (ii), and RNA-Seq data excluding genes lacking a corresponding probeset on the array (iii) were functionally characterized using the InterPro annotation (www.ebi.ac.uk/interpro). Information necessary to map the InterPro terms to the *Arabidopsis thaliana* gene identifiers was extracted from the functional gene descriptions available on www.arabidopsis.org (genes with no annotation were annotated as “unknown”). Some terms in the InterPro annotation are hierarchically linked to each other. Given this “parent to child” relation, a gene annotated with one term is automatically also annotated with all the ancestors of the term. To avoid reduction of statistical power due to this dependencies, we only used the lowest possible terms to characterize the genes. All terms annotating a gene were then grouped together, forming a specific combination of protein domains. To test for enrichment of a given combination in the RNA-Seq data, occurences were calculated and compared using Fisher's exact test (one-sided). Combinations with a p-value below 0.05 were declared to be significantly enriched (due to redundancies in the InterPro annotation, multiple testing correction may have been to stringent).

#### Genes preferentially expressed in central cells

The transcriptome of the central cell was compared to publicly available RNA-Seq transcriptome data from various tissues and cell types of *Arabidopsis thaliana*. The data comprised 2–4 cell and globular stage embryos [12] (GSE24198, GSE33866), early globular embryos [29] (SRR074122), whole plants (pool of organs) [18] (SRR018346, SRR018347, SRR019035), seedlings [18] (SRX006704), unopened flower buds [19] (SRX002554), and male meiocytes [30] (SRX063784). Raw data (csfasta/qual and fastq files) were downloaded from www.ncbi.nlm.nih.gov/geo/ (GSE accession numbers) and trace.ddbj.nig.ac.jp/DRASearch/ (SRX/SRR accession numbers). Only data from untreated wild-type plants were used in the analysis. The data was largely processed as described above with modifications depending on the experimental setup and without the thresholds of 5 hits and at least one hit in the first 250 bp of a transcript. In the data sets from [12] (50 bps reads, SOLiD), reads with multiple alignments were removed due to their high abundance (a consequence of the amplification strategy using random hexamers in addition to the poly(T)-primers for cDNA synthesis). For the remaining data sets from [18], [19], [29], [30] (36 bps reads, Illumina), reads could not be corrected and the allocation distance was set to 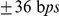. Genes preferentially expressed in central cells compared to all other tissues and cell types were then identified with edgeR [31] using tagwise dispersion estimates and Benjamini-Hochberg multiple testing corrections. Genes with an adjusted p-value (FDR) below 0.05 were considered to be differentially expressed. To test for enrichment of certain (combinations of) protein domains in the central cell transcriptome, we compared the functional characterization of the genes significantly upregulated in central cells with the one of the genes showing no significant differential expression using the approach described above (Table S4).

### Data processing: *de novo* assembly

Reads were corrected as described above. We removed all reads which were of low quality (total quality below 200 or an ambiguous color in the sequence), repetitive (same double color in more than 30% of the sequence), or duplicated. Assembly was performed on double encoded reads using velvet (version 1.0.18 [36]) and oases (version 0.1.18, www.ebi.ac.uk/ 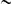
zerbino/oases) with a k-mer length of 31, a minimal transcript length of 80, and a minimal coverage of 1. Double encoding and decoding was done using the pre- and postprocessor scripts (versions 2.2.1 and 1.6, solidsoftwaretools.com/gt/project/denovotools) in conjunction with asid_light (version 1.0, solidsoftwaretools.com/gt/project/denovo). All reads were then mapped back to these assembled reference transcriptomes using bowtie with the options -C -n 2 -l 25 -k 11 -m 10 –chunkmbs 1024 –best –strata -p 7 (version 0.12.7; [37]). Ambiguous alignments were proportionally distributed using the number of unambiguous alignments. The final expression values were calculated as the sum of hits mapping to a transcript. Assembled transcripts and representative gene models from the reference annotation (www.arabidopsis.org) were annotated using Blast2GO (version 2.4.8; [38]). For blastx against the non-redundant protein sequences deposited at NCBI (www.ncbi.nlm.nih.gov) an e-value threshold of 1e-6 was chosen. Parameters for the GO annotation and analysis were left at default. To compare the annotations, we used the tool embedded in Blast2GO (Blast2GO version 2.4.9). GO terms with an FDR below 0.05 were defined as being significantly differentially enriched (two-sided Fisher's exact test).

### Microarray data

Microarray data [3] were obtained from ArrayExpress (www.ebi.ac.uk/arrayexpress, accession number E-MEXP-2227) and processed as described in [22]. Final expression values are given in Table S2.

### Software

Unless specified, we used newly developed software. The core package is split into several programs which are largely independent of each other (processing of reads with multiple alignments, filtering of genes, distribution of ambiguous hits, filter for *de novo* assembly) and therefore offers flexibility to customize and extend the analysis. Source code and linux binaries for the transcriptome analysis are freely available upon request (schmid.m@access.uzh.ch).

### MIAME

All data are MIAME compliant. Raw data were deposited at (RNA-Seq data: GSE29719 on GEO) and obtained from (microarray data: E-MEXP-2227 on ArrayExpress; RNA-Seq data: GSE24198, GSE33866, SRR074122, SRR018346, SRR018347, SRR019035, SRX006704, SRX002554, SRX063784 on GEO and DRASearch) MIAME compliant databases.

## Supporting Information

File S1 The file contains the results from the cDNA library control experiments (size distribution of fragments and approximate concentration of selected genes).
(PDF)

File S2 The rar file contains the transcript sequences from the *de novo* assembly (fasta file).
(RAR)

Figure S1 The figure contains a summary of the overlaps of the potentially new loci producing transcripts supported by splice junctions given in Table S1 between the two replicates.
(PDF)

Table S1 The table contains the genomic coordinates and annotations of the potentially new transcripts, which were identified and annotated using cufflinks and Blast2GO, respectively. Only transcripts supported by at least one splice junction are presented.
(XLS)

Table S2 The table contains the RNA-Seq expression values from all genes declared to be present in at least one of the replicates (sheet 1) and the microarray expression values from all the genes having a corresponding probeset on the microarray (sheet 2).
(XLS)

Table S3 The table contains the results from tests for enrichment of InterPro domains in the RNA-Seq data compared to the array data. In the first test (sheet 1), the gene universe was defined as the union of all the genes found to be expressed in any of the data type. In the second test (sheet 2), genes, which were present in the RNA-Seq data but are per default not detectable with the array due to the lack of a corresponding probeset, were excluded from the universe. The third sheet contains a table with additional information to Figure 5.
(XLS)

Table S4 The table contains the results from tests for enrichment of InterPro domains in the central cell transcriptome compared to transcriptomes of other tissues. The test set contained all genes showing significant enrichment in the central cell. The reference set comprised all the other genes (only the ones sequenced in at least one experiment).
(XLS)
