# Supplementary material for: A Powerful Method for Transcriptional Profiling of Specific Cell Types in Eukaryotes: Laser-Assisted Microdissection and RNA Sequencing
Source: PLoS One. 2012 Jan 26;7(1):e29685. doi: 10.1371/journal.pone.0029685 (PMC3266888; doi:10.1371/journal.pone.0029685)
Supplement: File S1 — The file contains the results from the cDNA library control experiments (size distribution of fragments and approximate concentration of selected genes). (PDF) [file pone.0029685.s001.pdf]

Distribution of the size of cDNA libraries

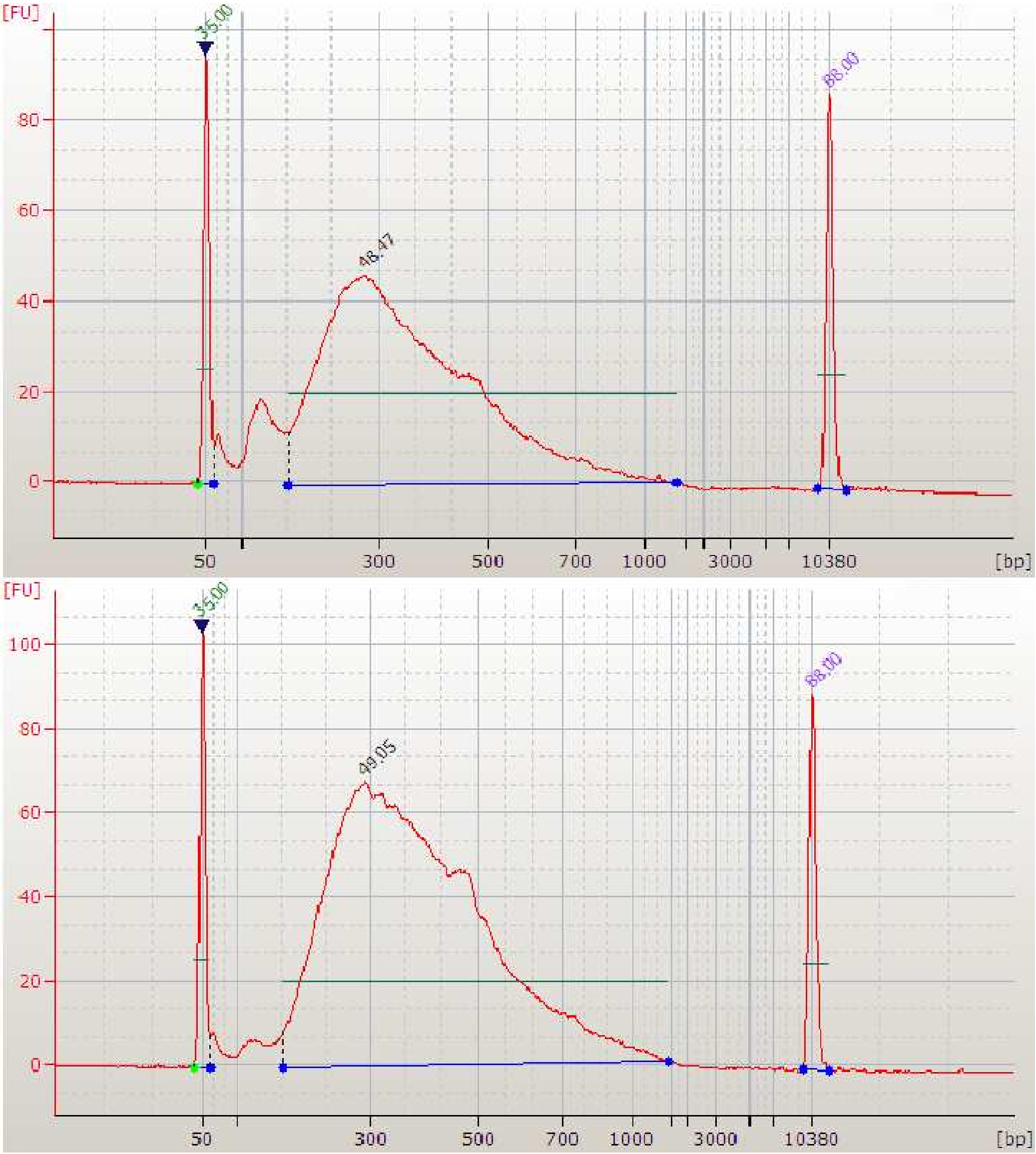

Figure 1: Size distributions of cDNA libraries were measured using Agilents Bioanalyzer (top panel: CC1, bottom panel: CC2). Sizes are given on the x-axis, arbitrary fluorescence units corresponding to the amount of fragments with the respective size on the y-axis. Peaks at the left and right end correspond to the size markers.

Quality control of the cDNA libraries (yield)

Table 1: Relative concentration of cDNA from selected genes in the cDNA libraries (water as negative control) was measured using qPCR. Given are the average Ct values from two technical replications. The value corresponds to the cycle at which the amplification product of the respective cDNA could be detected (lower value means higher relative concentration). In case no amplification product could be detected after 45 cycles, the value was set to "NA". The asterik marks the case where the amplification product could only be detected in one of the technical replicates.

gene	CC1	CC2	water
ACT2	28.1	25.8	36.0*
ACT11	31.4	29.4	NA
$EF-1\alpha$	27.8	24.9	NA
UBC9	26.0	24.4	36.8
