# Supplementary material for: A Powerful Method for Transcriptional Profiling of Specific Cell Types in Eukaryotes: Laser-Assisted Microdissection and RNA Sequencing
Source: PLoS One. 2012 Jan 26;7(1):e29685. doi: 10.1371/journal.pone.0029685 (PMC3266888; doi:10.1371/journal.pone.0029685)
Supplement: Figure S1 — The figure contains a summary of the overlaps of the potentially new loci producing transcripts supported by splice junctions given in Table S1 between the two replicates. (PDF) [file pone.0029685.s003.pdf]

Overlaps between potentially new loci with transcripts supported by splice junctions

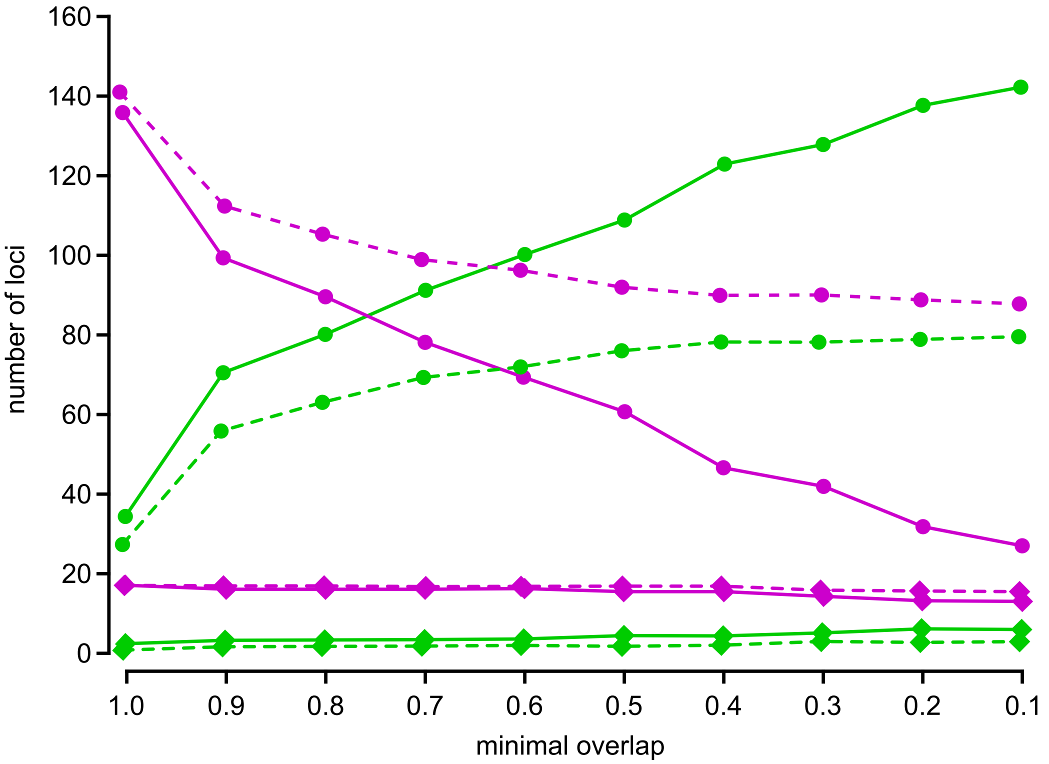

Figure 1: The figure summarizes the number of potentially new loci with transcripts supported by splice junctions that overlap to a given extent between the two replicates. For each locus, the overlap was defined as the ratio of the length of the regions with an overlap divided by the length of the locus. Thresholds from 0.1 (marginal overlap) to 1 (complete overlap) and the number of loci passing (green) or failing (magenta) the thresholds are drawn. Bullets for higher, diamonds for very low expression levels (less than 25 hits). Solid and dashed lines for the comparisons with and without the loci with transcripts not supported by splice junctions, respectively.
